# Early medical care and trauma management in mass casualties from major explosive accidents: a retrospective analysis and recommendations

**DOI:** 10.3389/fpubh.2025.1654156

**Published:** 2025-09-09

**Authors:** Zhengbin Wu, Jun Liu, Kaijing Xie, Diyou Chen, Qin Xiao, Shifeng Shao, Yaoli Wang

**Affiliations:** ^1^Department of ICU, Daping Hospital, Army Medical University, Chongqing, China; ^2^The Fifth Outpatient Clinic, Western Theater General Hospital, Chengdu, China; ^3^Department of Emergency Medicine, Daping Hospital, Army Medical University, Chongqing, China; ^4^Department of Radiology, Daping Hospital, Army Medical University, Chongqing, China

**Keywords:** blast injuries, mass casualties, triage protocol, damage control resuscitation, integrated trauma care system

## Abstract

**Objective:**

This study aims to analyze the clinical characteristics and treatment outcomes of mass casualties resulting from major explosive accidents and to identify the shortcomings in the management of such casualties during sudden explosive events.

**Methods:**

A retrospective analysis was conducted on data from a gas explosion at a cafeteria in a district of Southwest China. Key treatment measures, including injury assessments, early triage, timing of surgeries, hospital stay duration, and complications, were systematically collected and analyzed.

**Results:**

The explosion resulted in 16 fatalities at the scene and 10 survivors, all of whom received prompt triage, classification, transportation, and effective treatment. Among the survivors, two were male and eight were female, aged 26–50 years, with an average age of 39 years. The on-site mortality rate was 61.5%. Among the survivors, one patient with severe traumatic brain injury died 11 days post-incident despite medical intervention, while another was rehospitalized due to the recurrence of a pre-existing chronic condition. Three patients experienced varying degrees of hearing loss upon discharge, with one also reporting intermittent tinnitus. Four patients showed improvement and were discharged.

**Conclusion:**

The on-site mortality rate in mass casualties from major explosions is extremely high, and the injuries are often complex. Current early triage methods are limited and overly simplistic. The incidence of pulmonary injuries is high in explosive accidents, with rapid disease progression and elevated mortality. Timely identification of blast injuries and continuous monitoring of injury progression are critical to preventing further exacerbation of traumatic brain injuries and other organ damage. Strengthening regional trauma care infrastructure is essential for reducing preventable deaths.

## 1 Introduction

Large-scale casualties resulting from explosions continue to present a significant challenge globally, both for societies and healthcare professionals. Major explosive accidents are among the most severe emergencies to manage. From 1999 to 2006, the number of explosion incidents worldwide increased 4-fold, while the associated casualties rose 8-fold (1). The impact of such explosions on victims is profound, causing not only physical injuries but also severe psychological trauma. While non-military medical personnel have limited experience in managing blast injuries, as most explosion-related incidents occur in military settings, explosions can also result from industrial accidents and everyday situations, all of which require rapid medical intervention.

Blast injuries are categorized into five types based on their biomechanical mechanisms: Type I (primary blast injury), Type II (injuries from shrapnel, debris, or other projectiles), Type III (injuries from the explosion's shockwave, such as being thrown or crushed by collapsing structures), Type IV (injuries exacerbating pre-existing conditions or causing psychological trauma), and Type V (injuries due to bacteria, chemicals, or radiation associated with the explosion) (1, 2). Among these, primary blast injuries are particularly difficult to detect during early triage due to their often hidden nature, with symptoms sometimes appearing only after a delay (3, 4). The complexity of blast injuries extends beyond visible external trauma to include internal organ damage, such as to the lungs and intestines, which presents additional challenges in the initial assessment and management of these injuries (5).

Given the current situation in our country, mass casualties resulting from industrial and everyday accidents are the primary sources of blast injuries that healthcare personnel may encounter. These incidents also provide valuable data for clinical evidence-based medicine in China (6). In managing such emergencies, scientific and rational triage, along with standardized emergency medical procedures, are essential. These practices ensure the systematic execution of damage control resuscitation, treatment, and transportation for critically injured victims, thereby guaranteeing timely and effective care (7, 8). This paper summarizes the experiences of early triage, classification, transportation, and treatment of casualties from a recent explosion incident, aiming to offer evidence-based guidance for healthcare professionals handling victims of major explosive accidents.

## 2 Clinical data

### 2.1 Southwest China cafeteria gas explosion incident and rescue efforts

At approximately 12:10 p.m. on January 7, 2022, a suspected gas leak in a cafeteria in Southwest China led to the accumulation of natural gas in the kitchen, which ignited upon contact with an open flame, resulting in a powerful explosion and subsequent collapse of the building. The incident left 26 individuals unaccounted for. The building involved was a two-story brick and concrete structure with a floor area of ~70 square meters and a total building area of about 150 square meters, featuring prefabricated roof components. At the time of the explosion, several individuals were dining in the cafeteria. The following table lists the specific progress of the rescue (Table 1).

**Table 1:** Statistics of rescued people at different time points.

**Time**	**Cumulative number of rescued**	**Number of deceased**	**Number of undiscovered**
3:00 p.m.	9	0	17
6:00 p.m.	13	3	13
7:00 p.m.	15	6	11
8:00 p.m.	20	9	6
11:05 p.m.	26	16	0

### 2.2 On-site rescue operations

**Figure 1:**
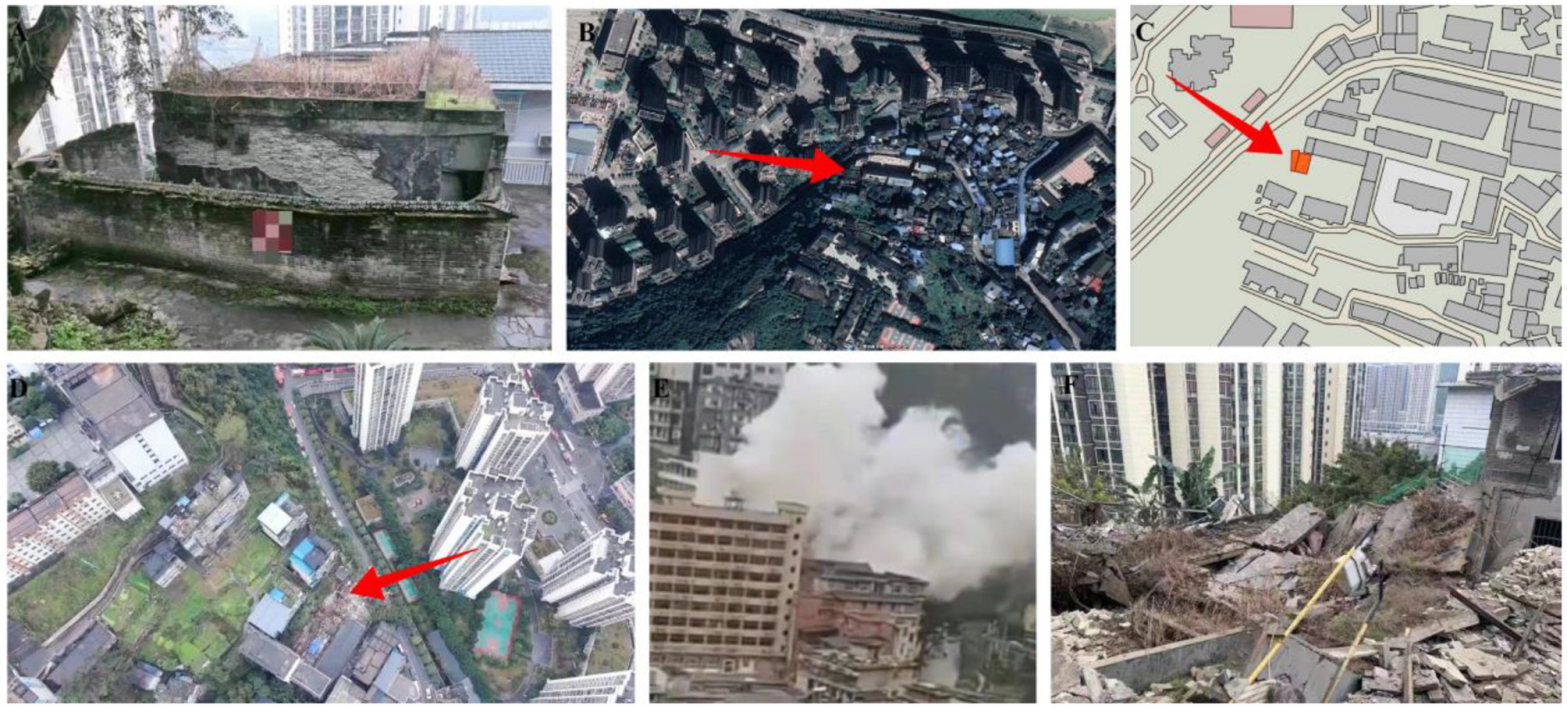
Explosion and on-site search, rescue, and treatment operations. **(A)** Condition of the building before the explosion. **(B)** Satellite picture of the building before the explosion. **(C)** The building location. **(D)** Drone aerial photo (after the explosion). **(E)** On-site conditions during the explosion. **(F)** On-site condition after the explosion.

Approximately 600 professionals participated in the rescue efforts, including 327 firefighters, 52 fire trucks, 60 members from mine and engineering rescue teams, and 20 pieces of heavy lifting and excavation equipment. In addition to the on-site search and rescue personnel, 19 medical professionals, specializing in trauma surgery, general surgery, neurosurgery, and intensive care, were involved in early field treatment and specialized transport. Six ambulances were deployed for patient transfer, successfully rescuing 26 individuals (Figure 1). Rescue workers used tools such as pickaxes, shovels, and steel bars to dig out and remove building materials to rescue the trapped people. Since the area where people were trapped was a small space of < 100 square meters with limited capacity, rescuers took turns to carry out the rescue work (Supplementary Figure 1). Medical personnel provided initial emergency care on-site and transported the injured to nearby hospitals for further treatment.

### 2.3 In-hospital treatment

Following the exhaustive rescue efforts, 10 survivors were identified, including 2 males and 8 females, aged between 26 and 50 years (average age: 39 ± 9.4 years). The injuries affected multiple areas, including the head, chest, abdomen, pelvis, and limbs. The injured were transferred to a nearby secondary hospital for detailed injury assessment and evaluation. Diagnostic measures included physical examinations, electrocardiographic monitoring, laboratory tests, and imaging studies. The severity of injuries and treatment plans were collectively assessed by a multidisciplinary expert team. Based on this assessment, patients were triaged to various specialties or the intensive care unit (ICU) for further treatment. Three patients with severe blast injuries underwent urgent damage control resuscitation and surgical interventions. Once stabilized, these patients were transferred to a tertiary medical center and, ultimately, to a level-four trauma center for continued care.

## 3 Treatment results

The explosion resulted in 16 fatalities and 10 injuries, yielding an on-site mortality rate of 61.5%. After receiving intensive treatment, one additional death occurred, bringing the overall mortality rate following treatment to 10%.

### 3.1 Injury characteristics

Due to the thick winter clothing worn by the victims, the explosion's projectiles were often unable to penetrate, resulting in injuries primarily to exposed areas of the skin, including abrasions, tears, and contusions. Basic physical examinations and monitoring of vital signs were sufficient for the rapid identification of Type II and III blast injuries. However, pulmonary injuries, cranial blast injuries, and other internal traumas were not immediately detectable through these initial measures. These patients required transfer to a secondary medical facility for further assessment and comprehensive evaluation of systemic injuries upon hospital admission (Tables 2, 3).

**Table 2:** General patient information.

**No**.	**Age (years)**	**Primary diagnosis**	**Blast injury type**	**Injury severity**	**Admission department**	**Length of stay (days)**	**Main surgical procedure**	**Referral**	**Prognosis**
1	40–50	1. Ear impact injury; 2. Multiple skin and soft tissue contusions	I	Mild	Orthopedics	13	Debridement and suturing	No	Paroxysmal tinnitus
2	20–30	1. Right ear impact injury; 2. Left tibial fracture; 3. Mild traumatic brain injury	I + III	Mild	Orthopedics	19	Left tibial fracture reduction + intramedullary nail fixation	No	Recovered and discharged
3	20–30	1. Ear impact injury; 2. Right 5th metacarpal fracture; 3. Mild traumatic brain injury	I + III	Mild	Neurosurgery	13	Debridement and suturing + foreign body removal	No	Recovered and followed up
4	30–40	1. Ear impact injury; 2. Multiple skin and soft tissue contusions with facial subcutaneous foreign body; 3. Left 2nd rib fracture	III	Mild	General surgery	13	Facial skin debridement and suturing + foreign body removal	No	Right ear hearing loss
5	40–50	Severe facial damage caused by heavy object impact	III	Severe	Intensive care	7	Tracheotomy	Yes	Recovered and discharged
6	40–50	1. Aspiration pneumonia; 2. Systemic lupus erythematosus lupus erythematosus	IV	Severe	Intensive care	/	Debridement and suturing + foreign body removal foreign body removal	No	Worsening of primary disease, continued hospitalization continued hospitalization
7	20–30	1. II° burns to face and both hands; 2. Right ear impact injury; 3. Pelvic fracture	I + III + IV	Severe	General surgery	14	Debridement and suturing	No	Bilateral hearing loss, right lower limb sensory impairment
8	50–60	1. Explosion-induced composite injury; 2. Multiple injuries	I ++ II + III + IV	Critical	Intensive care	37	Left thigh amputation + right lower limb debridement and skin graft	Yes	Bilateral hearing loss, right lower limb sensory impairment
9	50–60	1. Explosion-induced composite injury; 2. Multiple injuries	I ++ II + III + IV	Critical	Intensive care	11	Right femoral artery and vein exploration and anastomosis	Yes	Death
10	40–50	1. Multiple injuries (multiple fractures); 2. Thrombosis in the right lower leg	I + III	Severe	Intensive care	17	Debridement and suturing	No	Right lower limb sensory impairment, discharged

Systemic lupus erythematosus is the patient's underlying disease.

**Table 3:** Emergency initial diagnosis information.

**No**.	**HR (bpm)**	**R**	**SBP (mm Hg)**	**DBP (mm Hg)**	**SpO2^a^**	**RTS**	**ISS**
1	101	29	132	74	96%	15	4
2	96	29	121	79	95%	14	8
3	112	32	109	76	96%	13	6
4	121	34	137	82	98%	15	6
5	126	36	141	88	97%	10	25
6	127	36	130	77	83%	9	25
7	119	31	137	69	93%	14	16
8	142	41	82	54	93%	8	50
9	138	39	86	58	91%	5	57
10	114	31	100	59	97%	13	36

a These data were measured under nasal cannula oxygen inhalation or ventilator - assisted respiration, and the oxygen inhalation concentration was not collected.

### 3.2 Damage control surgery and resuscitation

Five patients with severe combined injuries from the explosion were transferred to a trauma center for treatment.

Patient 8, underwent emergency surgery under general anesthesia on January 7, 2022, for “left thigh amputation and right lower limb debridement and skin grafting.” During the surgery, he received 1,200 ml of red blood cell suspension and 800 ml of frozen plasma. Post-operatively, the patient was transferred to the ICU for damage control resuscitation, where he was administered 2,400 ml of red blood cell suspension, 3,000 ml of frozen plasma, and 20 units of cryoprecipitate.

Patient 9, underwent emergency surgery under general anesthesia for “multi-site burn wound debridement, right femoral comminuted fracture external fixation, right lower limb wound debridement and repair, knee joint skin grafting, and negative pressure wound therapy (VSD).” During surgery, a total of 13,500 ml of fluid (7,750 ml of crystalloids and 3,000 ml of colloids) and 5,100 ml of blood products (1,500 ml of crystalloids, 3,500 ml of colloids) were infused. Additionally, he received red blood cell suspension (5,200 ml), frozen plasma (4,800 ml), cryoprecipitate (50 units), and platelets (2 units). At 45 h after the injury, the patient underwent surgeries such as intracranial hematoma evacuation. After several multidisciplinary consultations and other interventions, the patient eventually died of multiple organ dysfunction and extremely high intracranial pressure 11 days later.

Patient 5, underwent emergency tracheotomy and catheterization on January 7, 2022, before being transferred to the ICU for damage control resuscitation, where she received 1,600 ml of red blood cell suspension and 1,200 ml of frozen plasma.

Patient 6, underwent emergency debridement and suturing on January 7, 2022, followed by transfer to the ICU for resuscitation, where she received 1,600 ml of red blood cell suspension and 1,200 ml of frozen plasma.

Patient 2, underwent “left tibial fracture reduction with intramedullary nail fixation” under combined spinal-epidural anesthesia on January 16, 2022.

### 3.3 Medical evacuation

**Figure 2:**
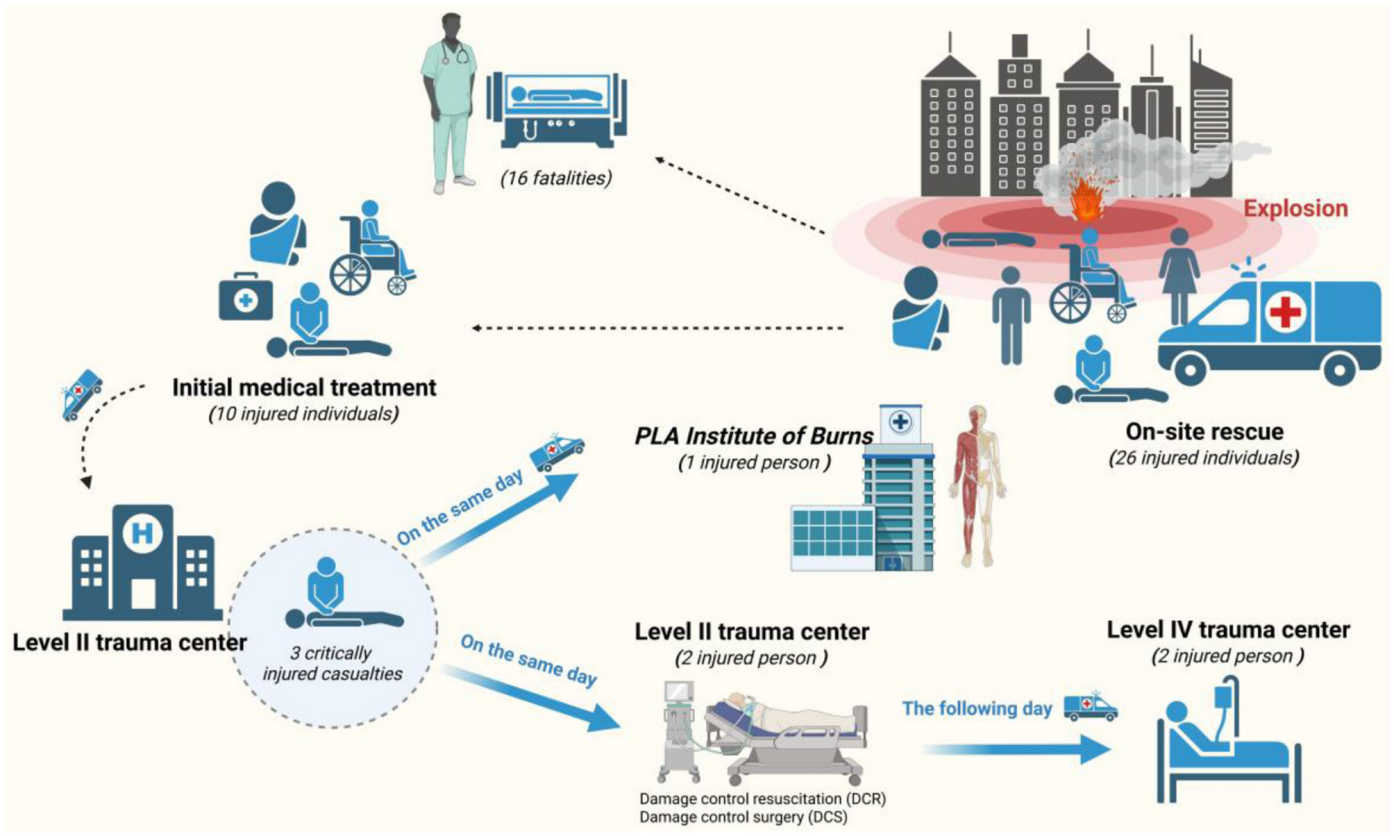
Flowchart for the treatment of explosion incidents.

A total of 26 people were rescued at the scene. After on-site triage, 16 people died, and 10 people were sent to a secondary trauma center after initial treatment. Among them, there were three severely injured patients: one was sent to the Military Burn Research Institute on the same day; two were sent to a quaternary trauma center the next day (January 8, 2022) after undergoing damage control resuscitation and damage control surgery at the secondary trauma center. The rest were not transferred (Figure 2). During transport, invasive mechanical ventilation was used for respiratory support, and continuous electrocardiographic monitoring was maintained to ensure patient safety.

**Figure 3:**
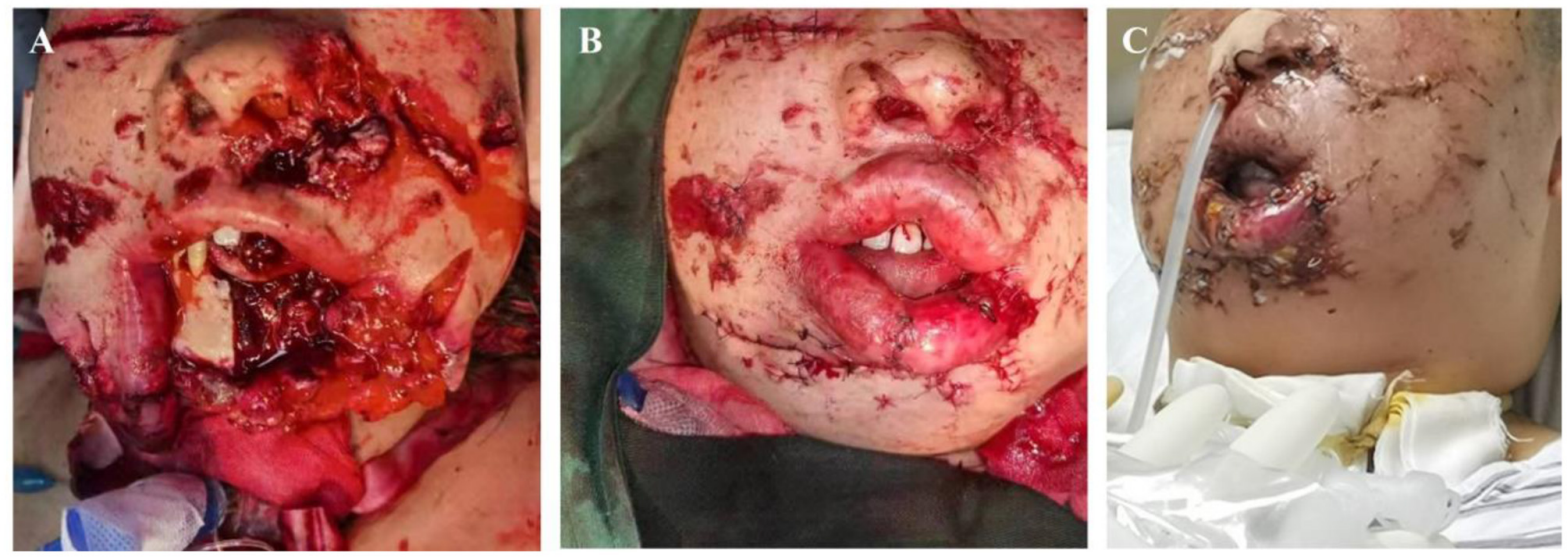
Severe Patient 5, diagnosis: injury to the jaw and face caused by a heavy object (ISS 36 points). **(A)** Jaw and facial injuries; tracheotomy to protect the airway. **(B)** Reconstructive surgery. **(C)** Post-reconstructive surgery.

**Figure 4:**
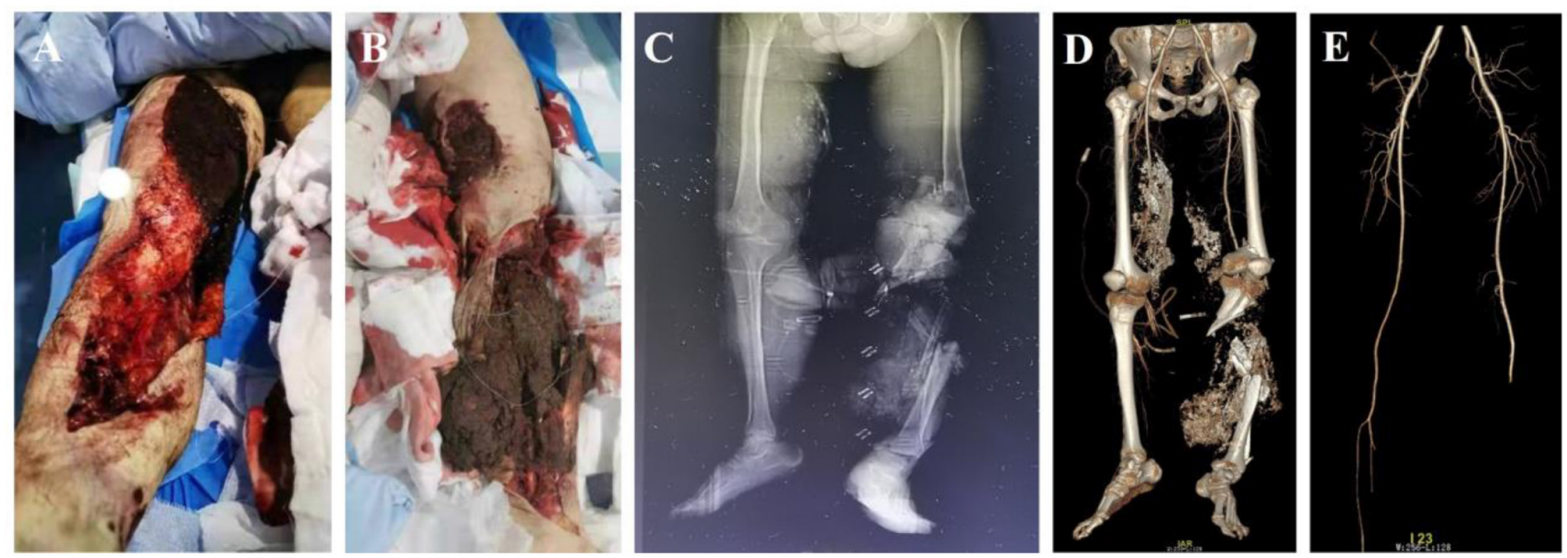
Critical Patient 8, diagnosis: blast-induced combined injury and multiple trauma (ISS 50 points: a tracheotomy and other surgeries were performed 35 h after the injury. Subsequently, damage control surgeries were conducted in 6 stages, and the patient was discharged after 37 days of treatment). **(A)** Open injury to the right lower limb. **(B)** Destructive injury to the left lower limb. **(C, D)**. X-ray and CT reconstruction of the pelvis and bilateral lower limbs reveals a comminuted fracture of the left tibia and fibula with vascular damage. **(E)** CTA of the bilateral lower limbs shows injury to the left calf artery.

**Figure 5:**
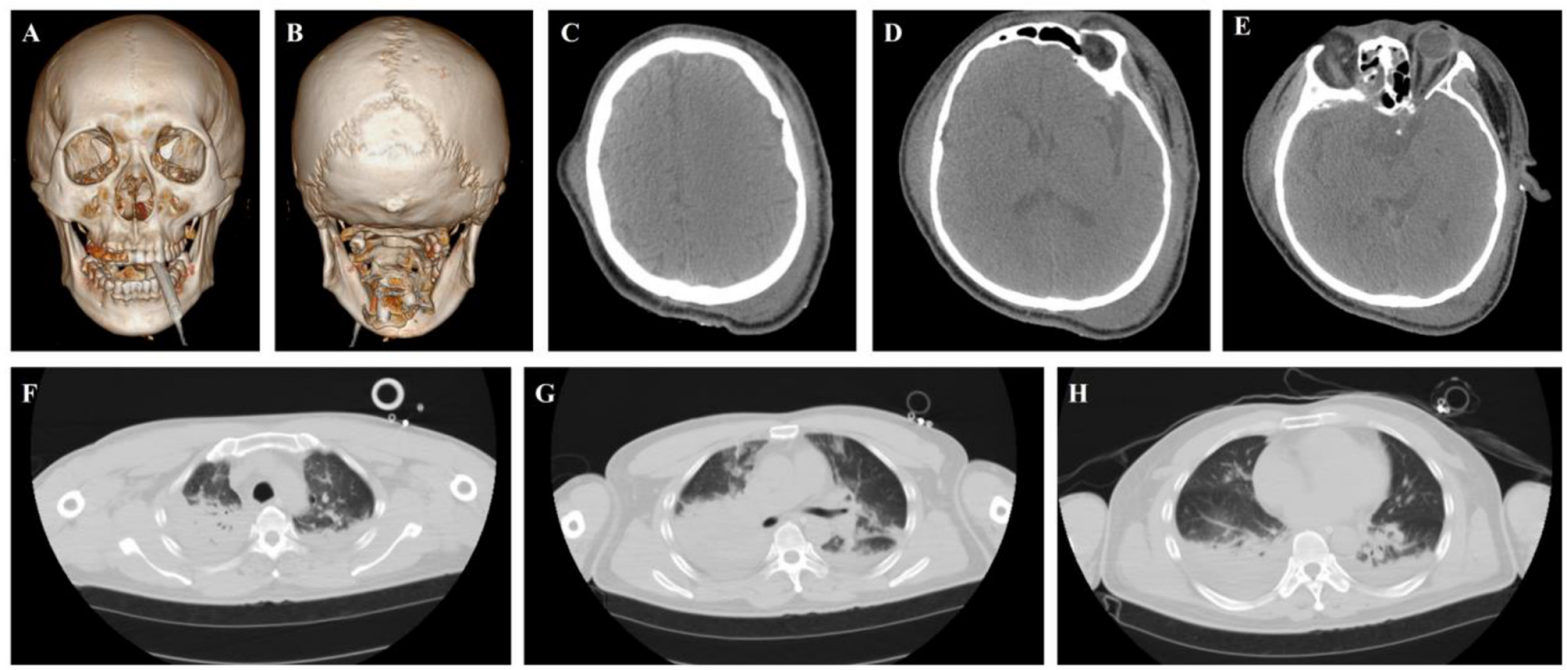
Critical Patient 8: cranial and thoracic imaging examinations. **(A–E)** CT of the head reveals mild cerebral edema. **(F–H)** Chest CT shows bilateral pulmonary infiltrates with consolidation, along with contusion.

**Figure 6:**
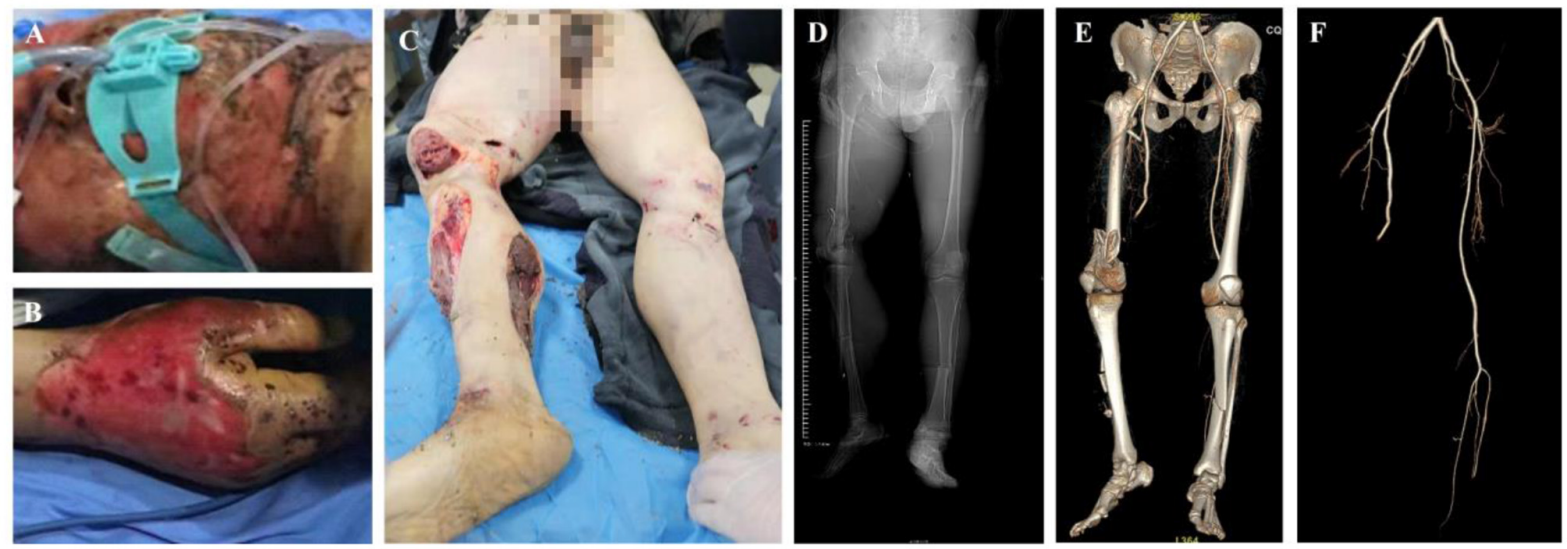
Critical Patient 9, diagnosis: blast-induced combined injury and multiple trauma (ISS 57 points: multiple-site surgeries, including amputation of the right thigh, were performed 35 h after the injury. Surgeries such as intracranial hematoma evacuation were conducted 45 h post-injury. Due to the critical condition, the patient was unable to undergo off-site imaging examinations. Subsequently, after several multidisciplinary consultations and two resuscitations, the patient died from uncontrollable intracranial hypertension and multiple organ failure on the 11th day after the injury). **(A)** Facial burns. **(B)** Hand burns. **(C)** Open fracture of the right lower limb. **(D, E)** X-ray and CT reconstruction of the pelvis and bilateral lower limbs reveals a comminuted fracture of the right distal femur and a mid-shaft fracture of the left tibia. **(F)** CTA of the bilateral lower limbs shows damage to the right femoral artery.

**Figure 7:**
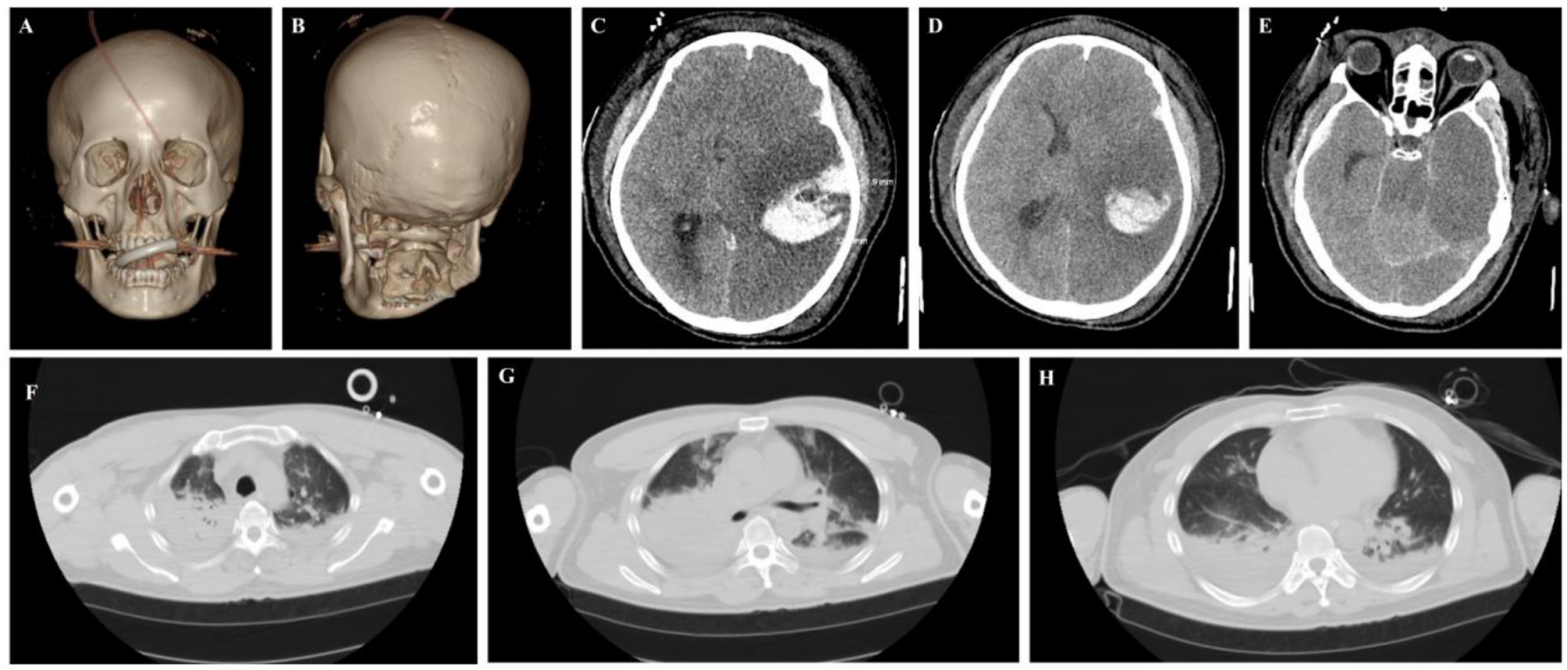
Critical Patient 9: cranial and thoracic imaging examinations. **(A–E)** Head CT reveals left temporal lobe hemorrhage with edema and compression of the lateral ventricle. **(F–H)** The chest CT scan showed bilateral lung exudation and consolidation, along with contusion.

Some patients with severe blast injuries experienced significant pulmonary congestion and edema due to the shockwave, necessitating high-pressure ventilation support (One of the patients had an inspiratory pressure of 20 cmH_2_O (above PEEP), with a peak inspiratory pressure below 27 cmH_2_O, a positive end-expiratory pressure (PEEP) of 7 cmH_2_O, and a plateau pressure of 23 cmH_2_O). Due to limited respiratory support equipment at the site, some patients developed hypoxemia in the early phase of treatment. After rapid on-site triage, the “appropriate respiratory support protocols” were specified to include specific methods such as oxygen pillow oxygenation, non-invasive ventilation, and emergency ventilator-assisted respiration. For patients with maxillofacial destructive injuries, individualized measures of “tracheotomy plus ventilator-assisted respiration” were supplemented. Additionally, severe vascular damage and aggressive fluid resuscitation often led to fatal hemorrhages, trauma-induced coagulopathy (TIC), and disseminated intravascular coagulation. These complications required urgent evaluation and intervention, followed by damage control surgery and systemic organ support. After initial field triage, three severely injured patients were transferred to a secondary medical facility for comprehensive evaluation and resuscitation, before being moved to a level-four trauma center and the Military Burn Research Institute for multidisciplinary consultations and staged surgical treatment. Ultimately, two patients were discharged alive, while one patient died despite extensive medical efforts (Figures 3–7 and Supplementary Figure 2).

### 3.4 Treatment outcomes

In the early treatment phase of severe blast injuries, management primarily focused on addressing airway congestion, high airway pressure, and hypoxemia resulting from low tidal volume. Treatment also included thrombolytic or anticoagulant therapy for deep vein thrombosis induced by TIC, as well as interventions for hypothermia, acidosis, coagulopathy (the “lethal triad”), and timely management of ventricular fibrillation. Among the 10 patients, 1 died on the 11th day post-injury due to sustained intracranial pressure (ICP > 163 mmHg). Eight patients were discharged after recovery, with three experiencing varying degrees of hearing impairment, and one reporting intermittent tinnitus. The average length of hospitalization for these eight patients was 19 ± 11 days. One patient required extended hospitalization for the recurrence of systemic lupus erythematosus triggered by the blast injury.

## 4 Discussion

A hazardous goods warehouse explosion in China in 2015 and a chemical company warehouse explosion in 2019 had significant social impacts, resulting in numerous casualties. Understanding the unique mechanisms of blast-induced trauma, the variations in organ damage, effective triage methods, and the availability of sufficient medical resources is crucial for healthcare personnel to manage mass blast injuries more effectively and ultimately save more lives. Both domestic and international research has shown that some blast injury patients experience delayed manifestations of injury (3–7). Therefore, pre-hospital triage methods and indicators that are simple, feasible, and prognostically relevant are particularly important in the early triage and classification of blast injuries, given the complex and multidimensional nature of the injury mechanisms (8).

### 4.1 Issues to consider in the treatment of severe blast injuries

Early triage and injury assessment are fundamental to trauma care, encompassing the primary survey, secondary survey, trauma scoring systems, assessment of sub-stable conditions, integration of clinical data, treatment sequencing, and evaluation of special injury types (9–11). The decision-making process regarding injury severity and treatment prioritization directly impacts the survival of critically injured patients, making it essential for the effective management of severe blast injury victims (12). Blast injuries are classified from primary through to quinary, reflecting the different mechanisms by which injury occurs, among the nine surviving patients we reported, most suffered from type I, II, and III blast injuries. Among them, Patient 6, who has systemic lupus erythematosus (SLE), is considered to possibly have concurrent type IV blast injury, that is, the exacerbation of pre-existing diseases caused by the explosion.

Primary blast lung injury and traumatic brain injury (TBI) can worsen within 24–48 h post-injury due to edema, potentially leading to life-threatening complications such as acute respiratory distress syndrome and brain herniation. Early identification and prompt management of these severe delayed injuries are crucial for improving survival outcomes (13–15). Given the limited rescue resources and challenging conditions at the explosion site, coupled with the complexity of injuries, intensive care plays a critical role in ensuring the safety and effective continuous treatment of severely injured victims (16). Among the critically ill patients, one required a relatively high inspiratory pressure to achieve a tidal volume of 6 mL/kg. This was considered likely due to poor lung compliance and airway edema, among other factors. Additionally, bronchoscopy and chest imaging examinations performed after the patient's admission confirmed the presence of lung injury. Transferring intensive care techniques to the pre-hospital phase—while considering injury severity, environmental factors, and treatment timeliness—can significantly improve survival rates. The treatment of the 10 critically injured patients in this incident tested the capacity of local medical teams for intensive care and emergency response. While coordination among intensive care units from different hospitals provided strong support, it also highlighted differences in approaches and techniques for managing severe blast injuries.

Understanding the compounding effects of multi-organ injuries in severe polytrauma caused by explosions is crucial for early management (17–20). For instance, limb ischemia can induce cardiovascular decompensation, while early precise triage and damage control strategies directly determine outcomes. Among these, assessing sub-stable conditions and evaluating surgical tolerance are particularly important.

Understanding the compounding effects of multi-organ injuries is crucial for the early management of severe, multi-trauma, and complex injuries caused by explosions (21). For instance, ischemic limbs can exacerbate cardiovascular complications, potentially becoming life-threatening (22). Effective early triage and scientifically planned treatment strategies directly influence patient outcomes. Within the trauma care system, recognizing sub-stable conditions and accurately assessing a patient's tolerance for damage control resuscitation surgery are particularly critical (23, 24).

### 4.2 Hearing impairment due to explosions

Tympanic membrane damage is one of the most common injuries among blast victims and is highly susceptible to shockwave impact (25, 26). Among the eight discharged patients, four exhibited varying degrees of hearing loss. Symptoms such as hearing impairment, tinnitus, and hyperacusis are frequently reported after blast exposure and can significantly reduce the quality of life. Moreover, tinnitus has been closely linked to cognitive disorders and depression, further exacerbating its impact on long-term wellbeing (27). The transportation of our casualties is mainly carried out by ambulance. It should be noted that for such injuries requiring helicopter rescue, jarring movements (e.g., takeoff and landing) could be detrimental to patient outcome.

### 4.3 Blast-related TBI

Blast injuries can result in severe head trauma through multiple mechanisms, often involving complex, combined injury patterns (28, 29). These mechanisms frequently lead to polytrauma and significantly contribute to blast-related mortality. Although blast-induced TBI is sometimes referred to as a “hidden wound,” its high fatality rate underscores the need for heightened clinical attention (30). Patient 9, a 50-year-old male, underwent a CT scan on the day of injury, which revealed severe closed head trauma, including a right temporal epidural hematoma, left occipital subdural hematoma, and a right temporal bone fracture. A follow-up CT scan the next day showed multiple brain contusions in the left frontal and temporal lobes, diffuse cerebral edema, and severe herniation. Additional hemorrhages were detected in both subdural spaces, the subarachnoid space, and the ventricular system. Right temporoparietal bone fractures and hemorrhagic involvement of the right mastoid region were also noted. Despite intensive medical intervention, the patient succumbed to severe blast-induced brain injury and elevated intracranial pressure (ICP) 11 days later. The pathological characteristics of explosive TBI remain an area of active research (31). Patient 8 sustained a mild traumatic brain injury, while Patient 9 suffered a severe traumatic brain injury. Despite undergoing surgery at an early stage, Patient 9 still died from uncontrollable intracranial hypertension and multiple organ failure. Due to its complex injury mechanisms and high rate of associated injuries, the clinical management of explosive traumatic brain injury must emphasize “rapid assessment, multidisciplinary collaboration, and full-course brain protection.” Early identification and intervention of life-threatening factors (such as intracranial hematoma, shock, and hypoxemia) are key to improving prognosis. Although modern diagnostic and treatment technologies have significantly increased the survival rate of patients, the disability rate among those with severe injuries remains relatively high.

### 4.4 Damage control resuscitation in patients with blast injuries

In damage control resuscitation for patients with blast injuries, besides vigilance against exacerbated traumatic coagulopathy caused by massive blood transfusion and fluid resuscitation, precise maintenance of hollow viscera and cerebral perfusion is also essential (32). Blast shock waves target gas-containing hollow organs (such as the gastrointestinal tract and lungs) through abrupt pressure gradient changes, and excessive fluid replacement can aggravate gastrointestinal edema and intra-abdominal hypertension, worsen pulmonary interstitial edema, and deteriorate oxygenation. Meanwhile, shock waves can directly transmit to the brain, causing blast-induced traumatic brain injury; both insufficient and excessive fluid resuscitation can impair cerebral perfusion pressure (CPP) and exacerbate brain damage (33, 34). Therefore, it is necessary to dynamically monitor the balance between mean arterial pressure and intracranial pressure, implement individualized fluid management in conjunction with cerebral oxygen saturation, and maintain multiple organ functions based on the specific pathophysiology of blast injuries to improve the success rate of treatment.

### 4.5 Strengthening the “integrated” trauma care system platform

The management of mass blast injuries in this incident revealed deficiencies in the construction and utilization of trauma information platforms. The effective treatment of mass casualties from major explosive accidents requires a well-integrated trauma care system that combines emergency management, trauma treatment, and disaster rescue operations (35, 36). In this case, the hospital's information systems—including pre-hospital triage and injury data collection—were tested under high-pressure conditions but ultimately ensured the integrity of emergency information, preventing data loss. The emergency information system significantly enhanced the accuracy of pre-hospital triage assessments, reduced triage times, and improved overall treatment efficiency (37, 38). These findings highlight the need to further strengthen trauma care networks and quality control information systems, ensuring interoperability between trauma care networks, the 120 emergency system, city-wide quality control meetings, and trauma center mapping. Establishing a hierarchical trauma treatment system at both city and district levels is essential, along with the development of a standardized trauma medical data platform. Additionally, integrating multiple level-four trauma centers into a unified trauma research network would facilitate collaborative advancements in trauma care. A patient-centered approach, aligned with emergency response timelines, should ensure seamless transitions between pre-hospital and in-hospital care. Real-time monitoring of treatment progress, early warning systems for severe injuries, and continuous evaluation of trauma care processes through clinical research will be crucial in improving trauma management quality at the district and county levels.

### 4.6 Integration of trauma care and emergency rescue systems

The effective integration of specialized trauma care teams across different trauma centers, along with the strategic use of the public transportation system to connect various levels of trauma facilities, can establish a robust network of emergency treatment bases. This system significantly enhances the capacity for stratified triage, classification, and rapid transport of mass casualties in large-scale emergencies (39, 40).

Among the 10 survivors in this incident, early identification of Type II and III blast injuries was more straightforward compared to primary blast injuries. Primary blast injuries involve unique pathophysiological mechanisms affecting hollow organs, often resulting in “mild external, severe internal” damage. Pre-hospital diagnosis of these injuries is particularly challenging and typically requires further in-hospital evaluation (41, 42). As a result, many patients only had their pulmonary and cranial shock injuries diagnosed after hospital admission. For critically injured patients, in addition to standard trauma care, close monitoring for severe pulmonary and cranial blast injuries is essential, as these injuries can rapidly progress to high-mortality complications if not promptly managed (43, 44). Delayed diagnosis and intervention increase the risk of adverse outcomes (45, 46).

Given the special conditions of the rescue site, which is located in a mountainous area with poor road access and limited space, we have focused on summarizing the core principles formed in practice: always give priority to treating patients with urgent respiratory and circulatory problems; promptly initiate the transfer process after preliminary on-site treatment; and continuously conduct multiple systemic assessments during transportation to dynamically monitor changes in the patient's condition. Upon admission, patients immediately undergo multi-site whole-body CT scans, with particular emphasis on organs vulnerable to blast injuries such as the lungs, intestines, and brain. Meanwhile, damage control surgery is performed, and strategies for optimizing circulatory support and maintaining respiratory function are further implemented.

The high on-site mortality rate observed in major explosive incidents underscores the critical role of integrating health emergency management with trauma care systems. The efficiency of organizational management and coordination plays a decisive role in improving patient survival rates (47, 48). However, this incident also revealed several deficiencies in early triage. First, pre-hospital data collection was challenging, with incomplete or inconsistent records limiting the availability of evidence-based guidance for early decision-making. Second, early triage methods were inadequate. Although non-invasive, portable triage tools—such as handheld ultrasound—are widely utilized in modern emergency medicine, their application in pre-hospital settings remains limited in China (49–51). While handheld ultrasound and multimodal monitoring devices are extensively used in hospital-based emergency trauma care, data supporting their effectiveness in detecting blast injuries in the pre-hospital phase remains insufficient, leading to delays in the identification of latent injuries (52, 53). Lastly, due to the relative rarity of explosive mass casualty incidents in China, medical personnel have limited hands-on experience with blast injuries. Most knowledge of shock injuries is derived from animal studies or literature reviews, which do not fully prepare clinicians for the life-threatening complications associated with shock injuries in real-world scenarios. This lack of direct experience negatively impacts the effectiveness of early triage and treatment strategies. Therefore, improving the organizational structure, treatment capabilities, and information platform for managing severe blast injuries is an urgent priority. Establishing an emergency medical rescue system tailored to the specific demands of mass casualty incidents from explosions, enhancing overall medical response capabilities. Meanwhile, unmanned aerial vehicle logistics systems should be introduced to ensure the rapid delivery of emergency medicines and blood products, and real-time diagnostic and therapeutic linkage between primary-level medical institutions and Grade A tertiary hospitals should be realized through teleconsultation platforms and training specialized personnel in blast injury management will be essential to improving outcomes in future disaster response efforts.

## Data Availability

The original contributions presented in the study are included in the article/Supplementary material, further inquiries can be directed to the corresponding author.
