# Supplementary material for: Early medical care and trauma management in mass casualties from major explosive accidents: a retrospective analysis and recommendations
Source: Front Public Health. 2025 Sep 9;13:1654156. doi: 10.3389/fpubh.2025.1654156 (PMC12454366; doi:10.3389/fpubh.2025.1654156)
Supplement: Supplementary file 1 [file Data_Sheet_1.docx]

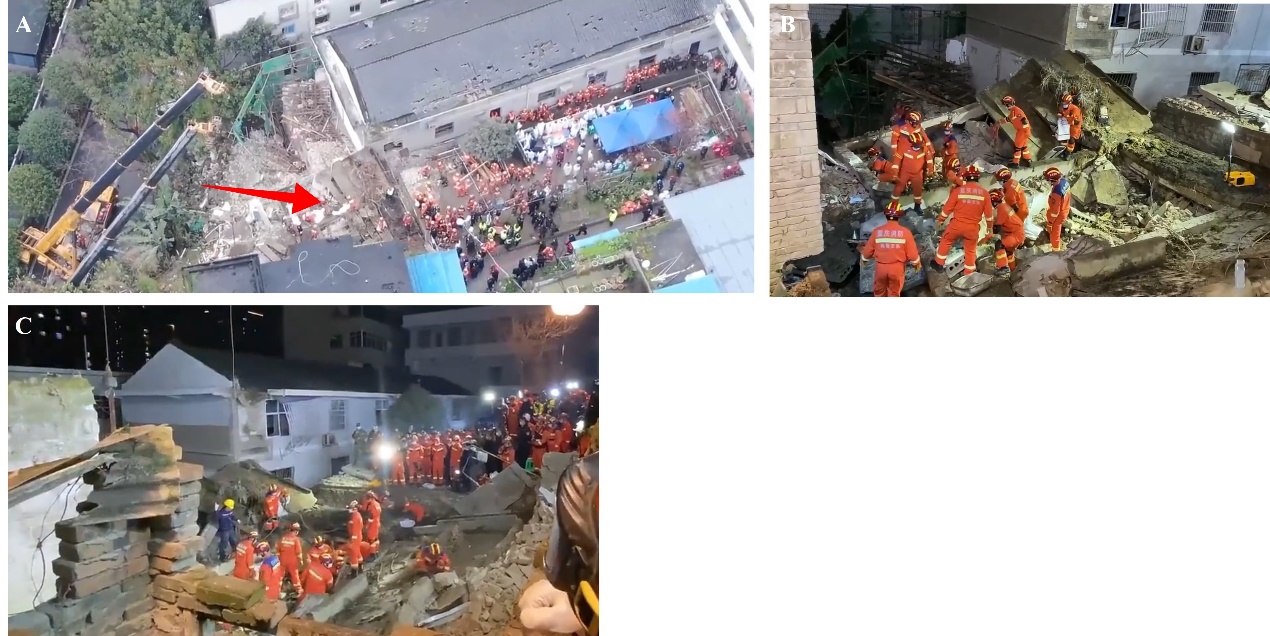


Supplementary Figure 1. On-site rescue.

(A)Aerial photos taken by drones of the on-site rescue after a building collapse. (B,C)On-site personnel search and rescue.
