# Supplementary material for: Early medical care and trauma management in mass casualties from major explosive accidents: a retrospective analysis and recommendations
Source: Front Public Health. 2025 Sep 9;13:1654156. doi: 10.3389/fpubh.2025.1654156 (PMC12454366; doi:10.3389/fpubh.2025.1654156)
Supplement: Supplementary file 2 [file Data_Sheet_2.docx]

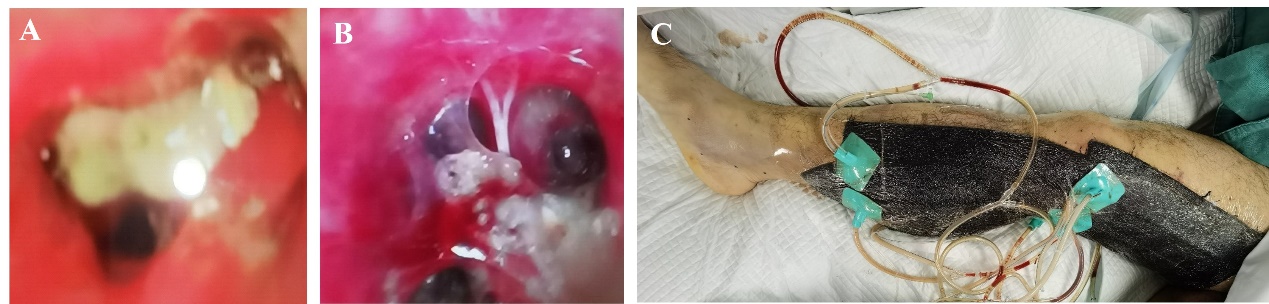


Supplementary Figure 2. Critical Patient 8, Diagnosis: Blast-induced combined injury and multiple trauma (ISS 50 points).

(A) Bronchoscopy on the first day post-injury shows airway congestion, edema, and secretion obstruction of the airway. (B) Bronchoscopy on the second day post-injury reveals reduced airway edema, with continued obstruction of the airway by secretions. (C) On the first day post-surgery, left thigh amputation and debridement and skin grafting of the right lower limb.
